# Onset of meso-scale turbulence in active nematics

**DOI:** 10.1038/ncomms15326

**Published:** 2017-05-16

**Authors:** Amin Doostmohammadi, Tyler N. Shendruk, Kristian Thijssen, Julia M. Yeomans

**Affiliations:** 1 The Rudolf Peierls Centre for Theoretical Physics, Department of Physics, University of Oxford, 1 Keble Road, Oxford OX1 3NP, UK; 2 Center for Studies in Physics and Biology, The Rockefeller University, 1230 York Avenue, New York, New York 10065, USA; 3 Department of Applied Physics, Eindhoven University of Technology, 5600 MB Eindhoven, The Netherlands

## Abstract

Meso-scale turbulence is an innate phenomenon, distinct from inertial turbulence, that spontaneously occurs at low Reynolds number in fluidized biological systems. This spatiotemporal disordered flow radically changes nutrient and molecular transport in living fluids and can strongly affect the collective behaviour in prominent biological processes, including biofilm formation, morphogenesis and cancer invasion. Despite its crucial role in such physiological processes, understanding meso-scale turbulence and any relation to classical inertial turbulence remains obscure. Here we show how the motion of active matter along a micro-channel transitions to meso-scale turbulence through the evolution of locally disordered patches (active puffs) from an ordered vortex-lattice flow state. We demonstrate that the stationary critical exponents of this transition to meso-scale turbulence in a channel coincide with the directed percolation universality class. This finding bridges our understanding of the onset of low-Reynolds-number meso-scale turbulence and traditional scale-invariant turbulence in confinement.

## Abstract (web summary)

Bacteria continuously inject energy into their surroundings and thus induce chaotic like flows, namely meso-scale turbulence. Here, the authors show that transition to meso-scale turbulence and inertial turbulence observed in pipes share the same scaling behavior that belongs to the directed percolation universality class.

Low-Reynolds-number turbulence is established through continuous energy injection from the constituent elements of an active fluid in many biological systems, including bacterial suspensions^1^,^2^,^3^,^4^,^5^,^6^, cellular monolayers^7^,^8^,^9^ or sub-cellular filament/motor protein mixtures^10^,^11^. Although the inertia is negligible (Reynolds number ≫1) in such systems, active turbulence is characterized by a highly disordered distribution of vortices^12^,^13^. However, meso-scale turbulence in living fluids possesses a characteristic vortex length scale, which distinguishes it from scale-invariant inertial turbulence^14^, and it is considered a new class of turbulent flow^2^,^12^,^15^.

Despite extensive implications for diverse fluid dynamical systems and more than a century of research, the transition from pressure-driven laminar flow to inertial turbulence in even the simplest geometries remains one of the major unresolved problems in fluid mechanics. Elaborate experiments have recently shed new light on the nature of this transition by measuring the decay and splitting of local turbulent domains/clusters (puffs) in pipe flows and have determined the critical Reynolds number—ratio of inertial to viscous forces—at which the transition occurs^16^,^17^. Short-range interactions between the locally turbulent puffs, which feed on surrounding laminar flow as an absorbing state, drive a continuous transition to a fully turbulent flow. Recent experimental evidence from channel and circular Couette flows^18^,^19^, together with direct numerical simulation studies and predator–prey models^20^, have provided evidence that the transition at the critical Reynolds number is characterized by the directed percolation universality class.

Strikingly, here we show that for a profoundly distinct class of turbulence at low Reynolds number, the transition in a channel can also be characterized by the emergence of puffs created by microscopic activity of biological fluids. Even for this low Reynolds number class of turbulent-like flows that apparently lacks a perfectly unique absorbing state, we find that the critical exponents correspond to the directed percolation universality class subject to a small non-ordering, conjugated and intrinsic field.

## Results

### Vortex lattice as an intermediate state

**Figure 1:**
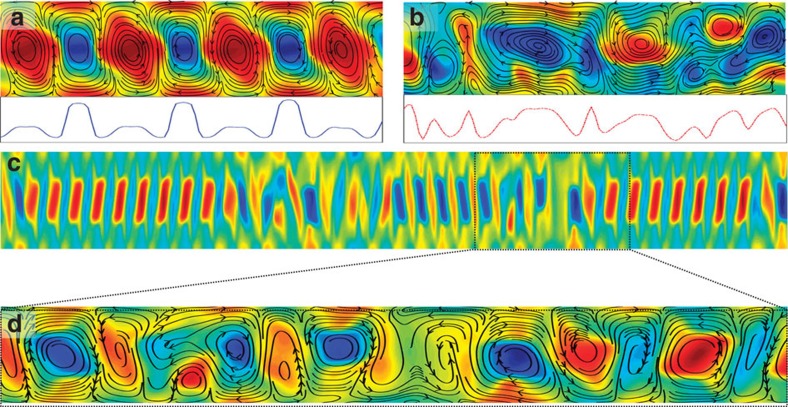
Emergence of puffs from a vortex-lattice controls the transition to active turbulence. (**a**) A highly ordered flow vortex lattice is formed at lower activities and (**b**) active turbulence is fully established at higher activities. Lower panels in **a**,**b** show the height-averaged enstrophy signal along the channel. (**c**) Coexistence of the vortex lattice and meso-scale turbulence close to the transition point. The zoomed-in panel in **d** illustrates the formation of active puffs from the vortex lattice. Colour maps show vorticity contours with blue and red colours corresponding to clockwise and anti-clockwise vortices, respectively. The average radius of vortices is 0.32*h*, where *h* denotes the channel height. Solid black lines illustrate streamlines of the flow.

**Figure 2:**
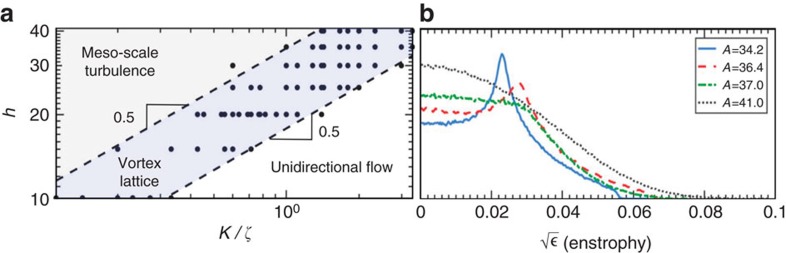
Competing length scales control the transition to active turbulence. (**a**) Phase-space of control parameters corresponding to the vortex-lattice state. The slope of 0.5 at the boundaries of the vortex-lattice state shows that the emergence of vortex lattice is controlled by two competing length scales: the channel height *h* and the activity length scale 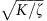. (**b**) Transition from the vortex-lattice state to active turbulence is characterized by the channel-averaged enstrophy 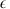 distribution for increasing values of activity number 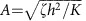.

To study the transition to low-Reynolds-number meso-scale turbulence, we computationally solve the continuum equations of active nematics in micro-channels, which have successfully reproduced the patterns of bacterial ordering in bulk^5^ and in confinement^21^, the flow structure and correlation lengths of microtuble bundles^10^,^11^,^22^ and the flow patterns of dividing cells^8^,^23^ (see Methods for the details of the model). Through this continuum description, the transition to turbulence occurs by increasing the amount of local energy injection (activity) in the living fluids. In a confined environment, the activity leads to spontaneous symmetry breaking and the generation of unidirectional flow^24^, which is followed by an oscillatory regime characterized by distorted streamlines^25^,^26^, upon increasing the activity. Further increase in the activity leads to the emergence of a stable lattice of vortices throughout the channel^27^ (Fig. 1a), and this transitions to meso-scale turbulence at higher activities (Fig. 1b). The emergence of the intermediate vortex lattice in active matter has been observed experimentally in motility assays of microtubles^28^, in bacterial suspension in a channel confinement^6^, and also numerically by short-range attraction of self-propelled particles^29^ and hydrodynamic screening of activity-induced flows due to frictional damping^30^. To focus on the effect of the confining channel on the transition to meso-scale turbulence, we consider the ideal ‘wet' limit and neglect additional frictional damping^11^,^31^,^32^. This is because in the experimental systems studied so far, there is no obvious qualitative effect on the active turbulence, friction appears to be a small effect, and the meso-scale turbulent state we consider here is unaffected^2^,^6^,^10^,^11^. We find that the intermediate vortex lattice is stable to impulsive perturbations, which do not produce growing modes (see Methods). In stark contrast to inertial turbulence, the Reynolds number is irrelevant here and the transition between flow regimes is governed by the dimensionless activity number 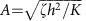 (Fig. 2a). This parameter characterizes the ratio of the channel height *h*, which here is equivalent to the hydrodynamic screening length, to the characteristic activity-induced length scale 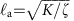, which represents the relative importance of the intrinsic activity *ζ* and the orientational elasticity *K* of the nematic fluid^33^,^34^.

The marked difference between the various flow states is clearly seen in the structure of the vorticity. Therefore, to characterize the transition between the regimes, we measure the distribution of the local enstrophy 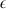, averaged across the channel. This quantity represents the strength of vortices in the flow, and has also been used for determining the nature of inertial turbulence. The vortex-lattice state possesses a well-defined peak in the enstrophy (Fig. 2b). As the active flow transitions at higher activities, the enstrophy distribution broadens, demonstrating that vorticity cascades down into meso-scale turbulence. The gradual disappearance of the peak in the enstrophy distribution (Fig. 2b) suggests a continuous transition from the vortex lattice to meso-scale turbulence. But how does the active turbulence develop from the vortex lattice?

### Critical behaviour

Figure 1c shows a snapshot of the vorticity field in a long channel close to the transition. The vortex lattice predominantly occupies the entire channel. Locally, however, we can identify regions of the channel where vortex pairs split into smaller non-ordered vortices (Fig. 1d). This coexistence of the global vortex lattice and clusters of local active turbulence controls the transition to turbulence in the channel. We term these localized domains of non-ordered vorticity active puffs, in analogy to the inertial puffs observed in the experiments on scale-invariant turbulence in long tubes^16^. Unlike the inertial puffs that are externally initiated by perturbations to the flow field (such as induced pressure jumps), active puffs are intrinsic to our simulations.

**Figure 3:**
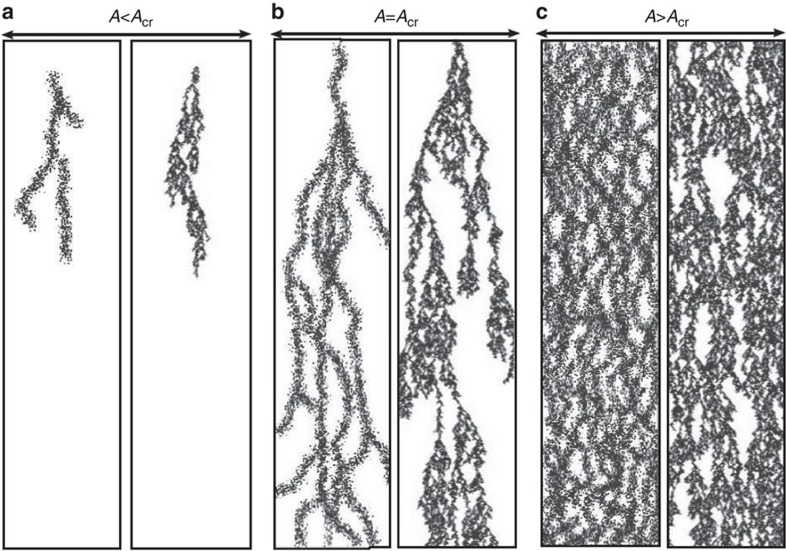
**Active turbulence percolates over time as the active puffs split and decay**. Spatiotemporal evolution of active puffs represented by space-time kymograph of the height-averaged enstrophy (**a**) below the critical activity *A*<*A*_cr_, (**b**) at the critical activity *A*=*A*_cr_ and (**c**) above the critical activity *A*>*A*_cr_. In **a**–**c**, left panels correspond to active turbulence and right panels show simulations from the Domany–Kinzel cellular automaton model of directed percolation model with rare spontaneous activations.

The behaviour of these active puffs is clearly characterized in the space-time kymograph of enstrophy (Fig. 3). An active puff can split, giving birth to new puffs, or decay into the ordered vortex-lattice state. Below some critical activity number, puffs tend to decay back to the inactive vortex-lattice state (Fig. 3a). On the other hand, active puffs span the entire system when splitting occurs at a high enough rate to produce a statistical steady state at the critical activity number (Fig. 3b). At higher activities yet, the competition between splitting and decaying of puffs results in a well-defined turbulence fraction within the channel (Fig. 3c). The active flow approaches the fully turbulent state with active puffs ultimately occupying the entire channel when the decay time far exceeds the splitting time^16^.

**Figure 4:**
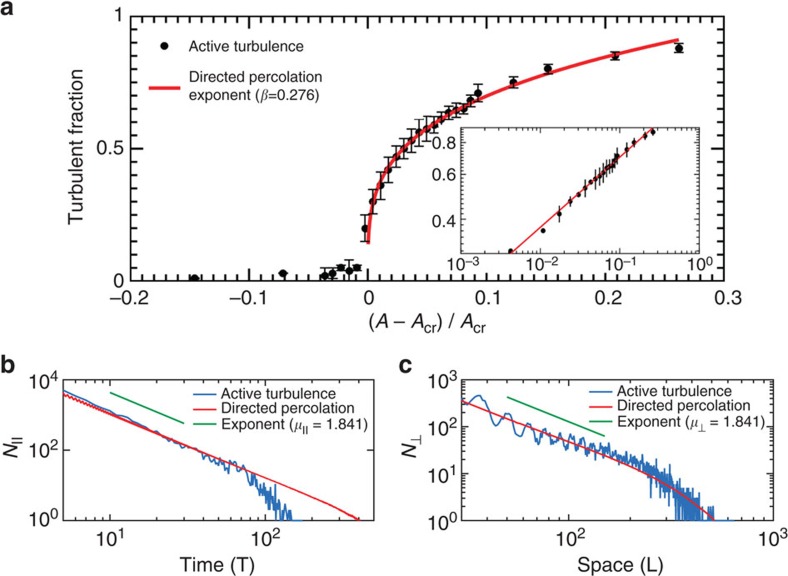
Transition to active turbulence coincides with directed percolation universality class. (**a**) Turbulence fraction as a function of the activity number. The red line corresponds to the turbulence fraction ∝(*A*−*A*_c_)^*β*^ with *β*=0.276 for (1+1) directed percolation with spontaneous activation. The error bars were calculated based on the s.d. from the mean value for simulations with different random initial conditions. Distribution of the vortex-lattice gaps is shown in **b** time and (**c**) space at the critical activity number. The green lines in **b**,**c** show *N*_||_∝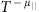, 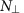
∝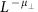, respectively with *μ*_||_=1.84,
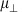
=1.748 for (1+1) directed percolation with rare spontaneous activations.

We thus measure the turbulence fraction, the area fraction occupied by active puffs in the channel, as a function of the activity number (Fig. 4a). Well below the critical point, active puffs have a short lifetime and rarely split (Fig. 3a), leading to a negligible turbulence fraction in the steady state (Fig. 4a). However, as the critical value of the activity is approached, puff decay becomes less likely and splitting time decreases substantially (Fig. 3b). Above the critical point, the puff population does not die out, producing a steady-state, non-zero turbulence fraction (Fig. 3c), and we find the turbulence fraction continuously increases with a power-law dependence ∼(*A*−*A*_cr_)^*β*^ (Fig. 4a). We measure the stationary exponent to be *β*=0.275±0.043, which closely matches the universal critical exponent of the (1+1) directed percolation process (*β*=0.276)^35^ and is in agreement with the value that has recently been measured for inertial turbulence in Couette flow (*β*=0.28±0.03)^19^.

This strong agreement is striking, as it draws a parallel between the low-Reynolds-number meso-scale turbulence in living fluids, which possesses a characteristic vorticity length scale, and high-Reynolds-number inertial turbulence, which is scale-invariant. Furthermore, the exponent is particularly surprising as active puffs are generated at a small activity-independent rate. In this way, the inactive vortex lattice is not a perfectly absorbing state, and yet there is remarkable agreement between the measured stationary exponent *β* and the critical exponent of the directed percolation universality class. The agreement with the universal critical exponent from directed percolation can be understood by recognizing that the creation of puffs corresponds to a weak, non-ordering field conjugated to the turbulence fraction, which is known to have no detectable effect on the stationary exponents sufficiently close to the critical point^36^,^37^.

To further scrutinize the critical behaviour at the transition point, we measure the spatial and temporal distributions of vortex-lattice gaps (see Methods). These distributions of the inactive state characterize correlations of the active puffs^38^ and obey power laws with exponents 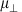, *μ*_||_ for space and time, respectively (Fig. 4b,c). The temporal exponent is measured to be *μ*_||_=1.84±0.04 and the spatial exponent is 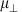
=1.8±0.1. These values also correspond to the critical exponents for (1+1) directed percolation (*μ*_||_=1.84,
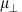
=1.748)^35^. The values of the exponents obtained from our measurements for meso-scale turbulence in a channel and for (1+1) directed percolation with spontaneous site activation are summarized in Table 1 and are compared with the experimentally measured exponents for the inertial turbulence in simple shear experiments in one-dimensional geometries^19^. It would be of interest to see if the critical exponents of the directed percolation universality class will continue to be found in geometries with higher effective dimensionality, as in experiments on inertial turbulence in quasi-two-dimensional passive liquid crystals^39^,^40^, in channel flows^18^ and in three-dimensional quantum turbulence^41^.

## Discussion

Our findings present a first concrete connection between turbulence in living fluids and classical scale-invariant turbulence, beyond a superficial visual similarity, by showing that the transitions to these two profoundly distinct types of spatiotemporal disorder in channel flows are characterized by the same scaling behaviour, corresponding to a critical directed percolation process. While the transition to scale-invariant turbulence corresponds to a critical absorbing phase transition, our results suggest that meso-scale turbulence is driven away from criticality by rare active puff creation that corresponds to directed percolation in the presence of a conjugated field. This opens new possibilities for further investigation of the nature of meso-scale turbulence and using tools from non-equilibrium statistical mechanics to explain transitionary behaviours in biological systems. Future research should investigate the transitions between ordered flow states, the nature of the weak conjugated field and the possibility of non-universal dynamical scaling behaviour or super-exponential dependence of puff lifetime on activity number.

## Methods

### Active nematohydrodynamics simulations

The spatiotemporal evolution of a living fluid is described by active nematohydrodynamics equations based on the theory of liquid crystals. This formulation has been extensively applied to biological systems including bacterial suspensions^21^, microtuble/motor protein mixtures^10^,^22^,^42^ and cellular monolayers^8^,^43^. The total density *ρ* and the velocity field *u* of the active matter obey the incompressible Navier–Stokes equations


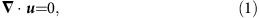



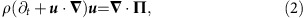


where Π is the stress tensor. While several studies of meso-scale turbulence have characterized the dynamics of the flow using only the velocity field as the relevant order parameter^2^,^15^, an additional order parameter field is required to account for the orientational order of active fluids. This is particularly important since several experiments have established the existence and pivotal role of the orientational order in the dynamics of bacterial suspensions^5^,^21^, microtuble bundles^10^,^11^, assemblies of fibroblast cells^44^ and more recently in stem cell cultures^9^. To account for the macroscopic orientational order of microscopic active and anisotropic particles, the nematic tensor 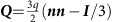 is considered, where *q* denotes the coarse-grained magnitude of the orientational order, ***n*** is the director and ***I*** the identity tensor. The nematic tensor evolves as


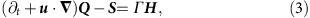


where *Γ* is a rotational diffusivity and the co-rotation term


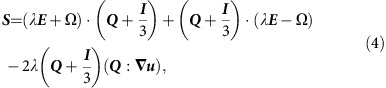


accounts for the response of the orientation field to the extensional and rotational components of the velocity gradients, as characterized by the strain rate ***E***=(**∇*****u***^T^+**∇*****u***)/2 and vorticity Ω=(**∇*****u***^T^−**∇***u*)/2 tensors, and weighted by the tumbling parameter *λ*. The relaxation of the orientational order is determined by the molecular field,


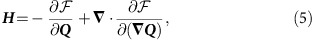


where 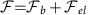 denotes the free energy. We use the Landau–de Gennes bulk free energy^45^,


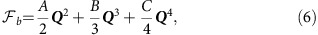


and 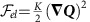, which describes the cost of spatial inhomogeneities in the order parameter, assuming a single elastic constant *K*.

In addition to the viscous stress Π^visc^=2*η**E***, equation (2) must account for contributions to the stress Π from the nematic elasticity and the activity. The nematic contribution to the stress is


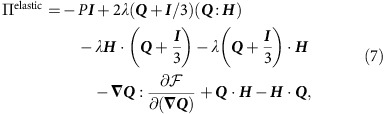


which includes the pressure *P* (ref. 46). The active contribution to the stress takes the form Π^act^=−*ζ**Q*** (ref. 47), such that any gradient in ***Q*** generates a flow field, with strength determined by the activity coefficient, *ζ*.

The equations of active nematohydrodynamics (equations (1, 2, 3)) are solved using a hybrid lattice Boltzmann and finite difference method^48^,^49^,^50^. We model a two-dimensional velocity field and nematic director^22^. While the director field can theoretically develop out-of-plane components, for active experiments and the parameter values used in this study, it does not move out of plane. Discrete space and time steps are chosen as unity and all quantities can be converted to physical units in a material-dependent manner^22^,^51^,^52^. Simulations are performed with the parameters *A*=0, *B*=0.3, *C*=−0.3, *Γ*=0.34, *K*=0.04, *λ*=0.3, *ρ*=1 and *μ*=2/3, in lattice Boltzmann units. Parameter fitting of the continuum equations to physical active systems remains a topic of research; therefore, we consider a generic parameter set that has been shown to reproduce the active turbulent state observed in bacteria and microtuble/motor protein suspensions^10^,^22^. With the largest observed average speed and vortex size, these parameters indicate a small Reynolds number Re≲*O*(10^−1^). Additional details can be found in refs 48, 49, 50, 53.

We use a channel with a height *h*=25 and length *L*=3,000. No-slip boundary conditions are applied to channel walls and periodic boundary conditions are used at the channel extremities. The results reported here are for strong homogeneous boundary conditions for the director field on the channel walls. In addition, we have performed simulations with homeotropic and weak anchoring boundary conditions and find that the transitions described in the main text are independent of the anchoring boundary conditions on the walls. To obtain sufficient statistics for the turbulence fraction (Fig. 4a), several instances (5–10) of each set of parameters with initially randomized fields are simulated. A warm-up of 3 × 10^5^ time steps is allowed to reach the stationary state. Above the critical point, this produces sample sizes of ≳100 puffs.

### Directed percolation model with spontaneous activation

To examine the behaviour of the (1+1) directed percolation universality class, we utilized a Domany–Kinzel cellular automaton^54^ and chose probabilities to correspond to site-directed percolation. This stochastic model is a discrete system on a diagonal square lattice of linear spatial size *L* with periodic boundary conditions. The state *s*(*i*,*t*) of site *i* at time *t* can be inactive (or empty) with *s*(*i*,*t*)=0, or the site can be activated (or occupied) *s*(*i*,*t*)=1 (ref. 35). In confined active nematic flows, the inactive state corresponds to the vortex-lattice state, and the activated phase corresponds to the active puffs of meso-scale turbulence. At time *t*, each site is occupied with some probability *P*_2_ if both backward sites (at time *t*−1) are occupied, and with probability *P*_1_ if only one backward site is occupied. Site-directed percolation is recovered with the choice *P*=*P*_1_=*P*_2_ (ref. 55). When the probability *P* reaches the critical probability *P*_c_, the system transitions from the absorbing phase of entirely inactive states to one in which the stationary density of active sites is non-zero and this transition is known to belong to the directed percolation universality class^35^.

In the confined active nematic, we find a small but non-zero rate of puff creation *P*_0_. Puff creation destroys the absorbing state, driving the system away from criticality. However, directed percolation in the presence of spontaneous site activation has been well studied, and it is known that the transition point is only weakly dependent on *P*_0_ (ref. 36), that spontaneous site activation is equivalent to a weak external field conjugated to the order parameter^35^, and that the stationary universal scalings of the directed percolation universality class continue to be found for sufficiently small spontaneous site activation (*P*_0_≲10^−3^)^56^.

Our directed percolation simulations employ periodic boundary conditions and a lattice size of 10^4^ sites in the spatial dimension to coincide with the lattice Boltzmann system. Data are obtained from 10^3^ runs of 5 × 10^3^ time steps each. We consider *P*_0_={0, 10^−9^, 10^−8^, 10^−7^, 10^−6^} and find the critical probability *P*_*c*_=0.64470±0.00002 as expected (0.6447001(1) (refs 35, 57)). We have found that the rate of puff creation does not depend on the activity coefficient *ξ*. Thus, at least near the critical point, there does not appear to be a suitable control parameter for *P*_0_. From the lattice Boltzmann simulations, we find that the rate of puff formation is (0.54±0.10) × 10^−7^∼10^−7^, which is the value used in the main text. Although *P*_c_ moves the system slightly away from the critical point, the directed percolation scaling exponents are not observed to change and are known to be independent of *P*_0_ in this weak field limit^32^. Measuring 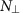 and *N*_||_, as for the lattice Boltzmann simulations, supplies the critical exponents reported in the main text.

### Calculating the turbulence fraction

**Figure 5:**
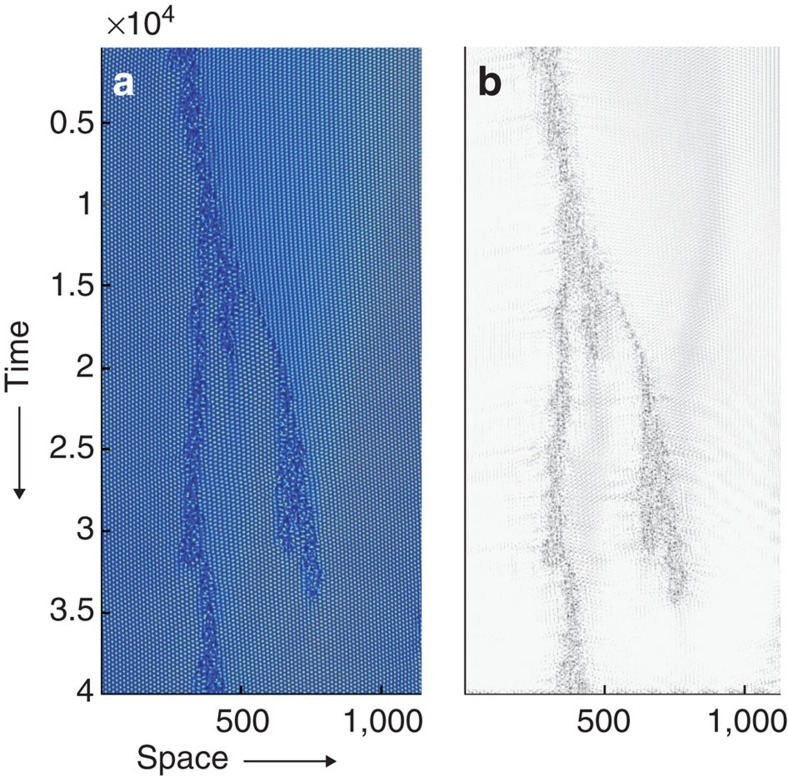
Masked images are produced to calculate turbulence fraction. (**a**) Unmasked and (**b**) masked images representing a sample kymograph of height-averaged enstrophy.

The enstrophy field *ɛ*(*x*, *y*, *t*)=Ω·Ω is calculated from the vorticity field Ω(*x*, *y*, *t*). The field is averaged across the channel 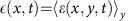 to create a 1+1 dimensional kymographical space-time signal. As described in the main text, the channel-averaged enstrophy in the vortex-lattice phase shows regular periodic oscillations, while local active turbulence domains (the active puffs) exhibit fluctuating, noisy enstrophy signals (Fig. 5a). To produce the visual traces of the active puffs (Fig. 3), the kymographs are Fourier transformed in both time and space. The primary peaks are masked in reciprocal space-time using Gaussian fits to produce the kymographs without the structured oscillations of the periodic background of the vortex lattice (Fig. 5b).

To quantitatively determine the turbulence fraction, the total simulation time is divided into *n* windows of size *τ*, creating a *L* × *n* array of space-time intervals, with each window *i* ranging from *t*_*i* × *τ*_ to *t*_(*i*+1) × *τ*_. Each of the temporal intervals is analysed for periodicity using a discretized temporal autocorrelation function 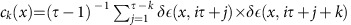, where *k* runs from 0 to *τ*, 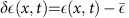, and 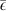 is the enstrophy signal averaged over the interval. While aperiodic signals decay to zero, oscillating functions possess periodic peaks. An interval is defined to be periodic if the peak amplitude exceeds a threshold value set by the 95 per cent confidence interval for a normal distribution with standard deviation *τ*^−1/2^. The time interval *τ* is chosen between three to five full oscillations of the vortex lattice, which is long enough to detect periodicity but minimizes the number of intervals containing both periodic and aperiodic regions. To reduce the possibility of false-positives, we recognize that the vortex unit cells have a finite size and duration. We filter the *L* × *n* kymograph of periodic/aperiodic windows, demanding that each periodic interval must possess at least one neighbouring periodic interval in time or space; otherwise, it is rejected.

The spatial interval distribution 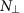 of the inactive state (the vortex lattice) is measured from the processed kymographs by recording the length intervals between active puff regions for fixed temporal coordinates. Similarly, the time interval distribution *N*_||_ between puffs is found by recording the temporal duration of the regions for fixed spatial coordinates. At short spatial intervals *L*, 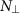 exhibits oscillations, which represent the characteristic size of the repeating vortex-lattice state.

### Assessing finite-size effects

**Figure 6:**
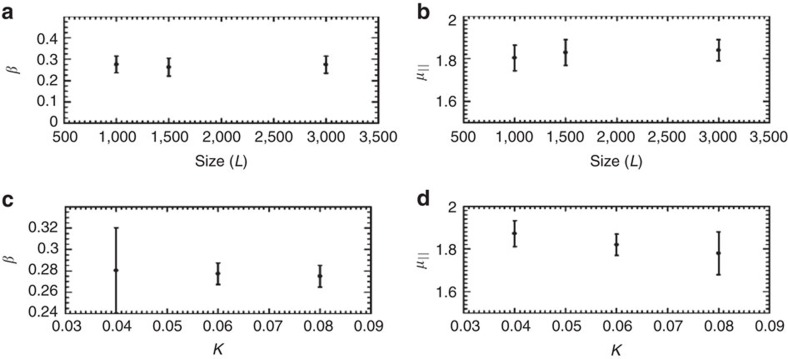
Finite-size and material parameters do not affect critical exponents. For system sizes smaller than those used in the main text (*L*=3,000), (**a**) the stationary critical exponent *β* and (**b**) the temporal correlations exponent *μ*_||_ are not found to be affected by finite-size effects to within uncertainty. (**c**,**d**) Likewise, the exponents are observed to be unaffected by increasing the nematic elasticity *K*. The error bars were calculated based on the s.d. from the mean value for simulations with different random initial conditions.

To test whether the critical exponents are affected by finite-size effects and physical parameters, we measure the critical exponents for various smaller lengths of the channel than those used in the main text, as well as increased nematic elasticity (Fig. 6). Within the uncertainty of the measurements, the stationary exponent *β* and the temporal correlations exponent *μ*_||_ are independent of these parameters.

### Stability of the vortex lattice

To probe the stability of the vortex lattice, the flow is subjected to instantaneous pulses at different times and locations along the channel. Even near the transition point, the perturbation to the mean enstrophy signal rapidly decays and the vortex lattice is not destroyed. The pulses only instantaneously shift the vortex-lattice configuration in space, and are not observed to grow into chaotic regions (active turbulence). Moreover, Fourier analysis of the perturbed enstrophy fields only exhibits the well-defined peaks associated with the vortex lattice and does not reveal growing modes that might indicate linear instability (below, at or above the critical point).

### Data availability

Source data and simulation materials are available from the authors upon request.

## Additional information

**How to cite this article:** Doostmohammadi, A. *et al*. Onset of meso-scale turbulence in active nematics. *Nat. Commun.* **8,** 15326 doi: 10.1038/ncomms15326 (2017).

**Publisher's note**: Springer Nature remains neutral with regard to jurisdictional claims in published maps and institutional affiliations.

## Figures and Tables

**Table 1:** Critical exponents for the transition to the meso-scale turbulence in a micro-channel.

**Critical exponents**	***β***	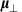	***μ***_**||**_
Active turbulence at low Reynolds number	0.275±0.043	1.80±0.10	1.84±0.04
Couette experiments for inertial turbulence^19^	0.28±0.03	1.72±0.05	1.84±0.02
(1+1) directed percolation^35^	0.276	1.748	1.84

Comparison to experimental measurements of inertial turbulence in Couette flow^19^ and directed percolation exponents^35^.
